# Effects of valproic acid on histone deacetylase inhibition in vitro and in glioblastoma patient samples

**DOI:** 10.1093/noajnl/vdz025

**Published:** 2019-11-12

**Authors:** Sharon Berendsen, Elselien Frijlink, Jèrôme Kroonen, Wim G M Spliet, Wim van Hecke, Tatjana Seute, Tom J Snijders, Pierre A Robe

**Affiliations:** 1 Departments of Neurology and Neurosurgery, UMC Utrecht Brain Center, University Medical Center of Utrecht, Utrecht, The Netherlands; 2 Department of Pathology, University Medical Center of Utrecht, Utrecht, The Netherlands; 3 Department of Human Genetics, GIGA Research Center, University of Liège, Liège, Belgium

**Keywords:** glioblastoma, histone deacetylase inhibition, in vitro, in vivo, valproic acid

## Abstract

**Background:**

The antiepileptic drug valproic acid (VPA) inhibits histone deacetylase in glioblastoma cells in vitro, which influences several oncogenic pathways and decreases glioma cell proliferation. The clinical relevance of these observations remains unclear, as VPA does not seem to affect glioblastoma patient survival. In this study, we analyzed whether the in vitro effects of VPA treatment on histone acetylation are also observed in tumor tissues of glioblastoma patients.

**Methods:**

The in vitro effects of VPA treatment on histone acetylation were assessed with immunofluorescence and western blotting. On tissue microarrays and in fresh-frozen glioblastoma tissues we investigated the histone acetylation patterns of patients who were either treated with VPA or did not receive antiepileptic drugs at the time of their surgery. We also performed mRNA expression-based and gene set enrichment analyses on these tissues.

**Results:**

VPA increased the expression levels of acetylated histones H3 and H4 in vitro, in agreement with previous reports. In tumor samples obtained from glioblastoma patients, however, VPA treatment affected neither gene (set) expression nor histone acetylation.

**Conclusions:**

The in vitro effects of VPA on histone acetylation status in glioblastoma cells could not be confirmed in clinical tumor samples of glioblastoma patients using antiepileptic doses of VPA, which reflects the lack of effect of VPA on the clinical outcome of glioblastoma patients.

**Importance of the Study**

The antiepileptic drug valproic acid (VPA) has been shown to inhibit histone deacetylase in glioblastoma cells, leading to the differential expression of several oncogenic pathways and decreased cell proliferation in vitro. Clinically, however, VPA does not seem to affect patient survival. In this study, we demonstrate that the in vitro effects of VPA on histone acetylation status do not occur in clinical glioblastomas in patients treated with antiepileptic dosages of VPA. This finding is a serious argument against (trials on) the use of VPA as an antitumor treatment for glioblastoma patients.

**Key Points**

1. In vitro VPA effects on histone acetylation in glioblastoma (GBM) are not observed in tumor samples from patients.
2. VPA affects neither gene expression nor histone acetylation in patient GBM tissues.
3. Lack of effects in tumor tissue reflects the lack of cytotoxic efficacy of VPA.

Glioblastoma (GBM) is the most aggressive primary brain tumor, and confers a dismal prognosis with a median survival of 15–20 months,^1^ despite extensive treatment. Genetic and epigenetic events condition the deregulation of growth, invasion, and therapeutic resistance pathways in GBM.^2,3^

Valproic acid (VPA) is a potent inhibitor of histone deacetylase (HDAC) and modulator of epigenetic events that has been used for several decades as an antiepileptic drug (AED) and mood stabilizer.^4^ VPA is also reported to elicit various antitumor effects in glioma cells in vitro.^5–8^ Through the inhibition of a subset of HDACs^4,9^ and cellular kinases (eg, GSK-3β),^10,11^ it decreases the activity of several transcription factors and signaling cascades, for instance NF-κB, STAT3, p53, p21, and Wnt/β-catenin signaling. In addition, in experimental gliomas, VPA reduces angiogenesis and inhibits tumor invasion, inhibits DNA repair, and potentiates the effect of chemotherapies (eg, temozolomide and etoposide) and radiation therapy (for a review, see Berendsen et al.^5^).

The study of large cohorts of patients with GBMs did, however, not find any favorable effect of VPA on GBM patient survival,^12,13^ especially when taking into account that epilepsy at diagnosis is a favorable independent prognostic factor, irrespective of AED use, in these tumors.^13^ This raises the question whether the clinical doses of VPA are able to elicit antitumor effects in GBMs in the clinical setting.

In order to answer this question, we compared the histone acetylation and RNA expression profiles of patient-derived GBM tissue samples obtained from patients treated or not with this drug.

## Methods

### Ethical Statement

This study was conducted following approval by the ethical committee, biobank, and institutional review board of the University Medical Center Utrecht (protocols 09-420, 14-225, and WARB2011/25, respectively).

### Patient Selection

Fresh-frozen GBM tissue was obtained from surgery, after written informed consent of the patients. Seventy-six fresh-frozen surgical samples of de novo GBMs were prospectively collected between 2010 and 2015, as reported previously.^14^ From these patients, we selected all patients that were treated with VPA for epileptic seizures at the time of surgery (*n* = 12). As a control group, we selected all patients that had experienced tumor-related epileptic seizures, but were not treated with any AED at the time of surgery (*n* = 7), as epileptogenic GBMs are biologically different from GBMs that do not cause epilepsy.^14–17^ These fresh-frozen samples were used for mRNA expression analyses and western blotting, as described later.

Archival fresh-frozen paraffin-embedded GBM tumor tissues from a consecutive cohort of 286 patients treated in the University Medical Center of Utrecht between 2009 and 2013 were included on tissue microarrays (TMAs) as described previously.^13^ Among these patients, we selected all patients receiving VPA for epilepsy at the time of their surgery. The control group consisted of all consecutive GBM patients with epilepsy who did not receive antiepileptic treatment at the time of their surgery.

The median VPA treatment duration until surgery was 33 days (range 13–196). In 11 patients, VPA treatment duration could not be verified. All patients on VPA continued the use of VPA in the perioperative timeframe.

IDH1 mutational status was not yet routinely determined in clinical practice at our center, but was available for 41 of 43 patients from a post hoc analysis from another study.^13^

### mRNA Expression Analysis

RNA was extracted with the Nucleospin TriPrep (Macherey-Nagel) and the QIASymphony RNA (Qiagen) kits according to the manufacturers’ instructions. Affymetrix HG U133 plus 2.0 arrays were prepared and scanned according to the manufacturer’s protocol and as reported previously.^18^

Differential gene expression analyses and exploratory gene set enrichment analyses (GSEA) were performed after Robust Multi-array Average (RMA) normalization^19^ and batch correction, with the Partek Genomics suite platform (v 6.6). Analyses were performed with the Broad Institute MySig libraries of curated gene sets C1–C7 version 5.0,^20^ 1000 permutations and default additional parameters. An false discovery rate (FDR) threshold of 0.1 was applied.

### Class Prediction

Molecular subclassification (proneural, neural, classical, mesenchymal) was determined by hierarchical clustering.^21^ Microarray normalization, data filtering, and analysis of inter-array homogeneity were performed as reported previously.^21,22^ Affymetrix HG U133 plus 2.0 probesets were matched to 840 genes originally published for the classification of GBMs (http://tcga-data.nci.nih.gov/docs/publications/gbm_exp/). Relative gene expression values were calculated. Genes were then excluded for a median absolute deviation below 0.5^21^. After filtering, 768 genes were used for the class prediction. The hierarchical clustering of samples was performed with cluster3 software^23^ with the agglomerative average linkage for the structure and 1 minus the Pearson’s correlation for the distance metric.^21^ Classes could be assigned to 17 of 19 samples.

### Cell Culture

Primary cell culture GM3 was obtained by mincing fresh surgical tumor samples as described previously.^24^ The U87 cell line was obtained from ATCC. Cells were maintained at low passages and cultivated in Dulbecco’s modified Eagle’s medium supplemented with 10% fetal bovine serum and 1% sodium pyruvate.

### Cell Survival Assay

To assess acute cell toxicity due to VPA treatment, cell survival in response to VPA treatment was assayed using an MTS test (One Solution Cell Proliferation Assay; Promega). Cell line U87 and primary cell cultures GM3 were treated with different concentrations of VPA (0, 0.1, 0.5, 1, 2.5, 5, and 10 mM) for 24 hours before performing the MTS assays. The MTS assays were performed as recommended by the manufacturer’s protocol.

### Western Blotting

Cells were treated once daily with VPA (0–1 mM) for 48 hours. We performed protein extraction with RIPA buffer (Sigma-Aldrich) or a nuclear extraction kit (Active Motif) and proteins were separated by means of 10% polyacrylamide gel electrophoresis. Membrane transfer was performed with the Trans-Blot Turbo Transfer System (Bio-Rad). After blocking of aspecific sites, membranes incubated overnight at 4°C in the presence of primary antibody directed against anti-histone H4 (acetyl K8) (rabbit polyclonal; Abcam), anti-histone H3 (acetyl K9) (rabbit polyclonal; Abcam), GAPDH (Rabbit; Sigma-Aldrich). An HRP-coupled secondary antibody was subsequently incubated, and the signal was developed with enhanced chemiluminescence (ECL) and the Super Signal West Femto Chemiluminescent substrate (Thermo Fisher Scientific) and the ImageQuant LAS 4000 Biomolecular Imager (GE Healthcare). Western blots were performed at least three independent times. We quantified the images with the gel analysis tool of ImageJ software (Rasband W.S., National Institutes of Health, Bethesda, Maryland, USA).

### Immunocytochemistry

Immunocytochemistry was performed as described previously.^25^ Cells were treated once daily for 48 hours with 0–1 mM VPA. Primary antibody incubation (anti-histone H3 (acetyl K9) (rabbit polyclonal; Abcam) and anti-histone H4 (acetyl K8) (rabbit polyclonal; Abcam)) was performed overnight at 4°C. RRX- and FITC-conjugated (Jackson ImmunoResearch Laboratories) were used as secondary antibodies. Omission of primary antibodies resulted in a complete loss of detectable signal.

Imaging was performed with use of a laser-scanning confocal microscope (Olympus Fluoview 1000). Microscope settings were kept constant across the experimental conditions. We quantified the fluorescence signal intensity per cell with ImageJ.

### Tissue Microarrays and Immunohistochemistry

A senior clinical neuropathologist reviewed histology of each specimen and performed marking on hematoxylin and eosin–stained sections. We reported details on TMA construction and immunohistochemical staining previously.^13^ The following primary antibodies were used: anti-histone H4 (acetyl K8) (Rabbit polyclonal; Abcam) and anti-histone H3 (acetyl K9) (Rabbit polyclonal; Abcam). The signal was developed with 3,3′-diaminobenzidine. Nuclei were counterstained with hematoxylin.

Researchers were blinded to the clinical data during evaluation of the protein expression. The percentage of nuclear staining and/or cytoplasmatic staining was scored as follows: 0, no positive staining; 1, 1%–25% positive staining; 2, 26%–50% positive staining; 3, 51%–75% positive staining; and 4, 76%–100% positive staining. A mean staining score was computed for each patient.

### Western Blotting Fresh-Frozen Tissues

Fresh-frozen GBM tissue from 14 patients (VPA: *n* = 8, no AED: *n* = 6) was available for western blotting. Protein extraction was performed with RIPA buffer containing protease and phosphatase inhibitors. Fifty nanograms of protein lysate were used for western blotting, which was performed as described earlier. We used the following primary antibodies: anti-histone H4 (acetyl K8) (rabbit polyclonal; Abcam), anti-histone H3 (acetyl K9) (rabbit polyclonal; Abcam), GAPDH (Rabbit; Sigma-Aldrich).

### Statistical Analysis

Statistical analyses were performed with Graphpad Prism 7 software. We checked the data distribution graphically with boxplots. The MTS test was analyzed compared to the control condition with one-way ANOVA with Dunnet’s post hoc test. The immunofluorescence data were analyzed compared to the control condition with a one-sample *t* test. Western blots with multiple dosages of VPA were analyzed with a two-sided ANOVA to control for interexperimental variation. The western blotting experiment containing patient material and immunohistochemistry results were analyzed with a Mann–Whitney *U* test. Two-sided *P*-values < .05 were considered significant.

## Results

### VPA Treatment Increases Expression of Acetylated Histone H3 and H4 in GBM Cells In Vitro

**Fig. 1.**
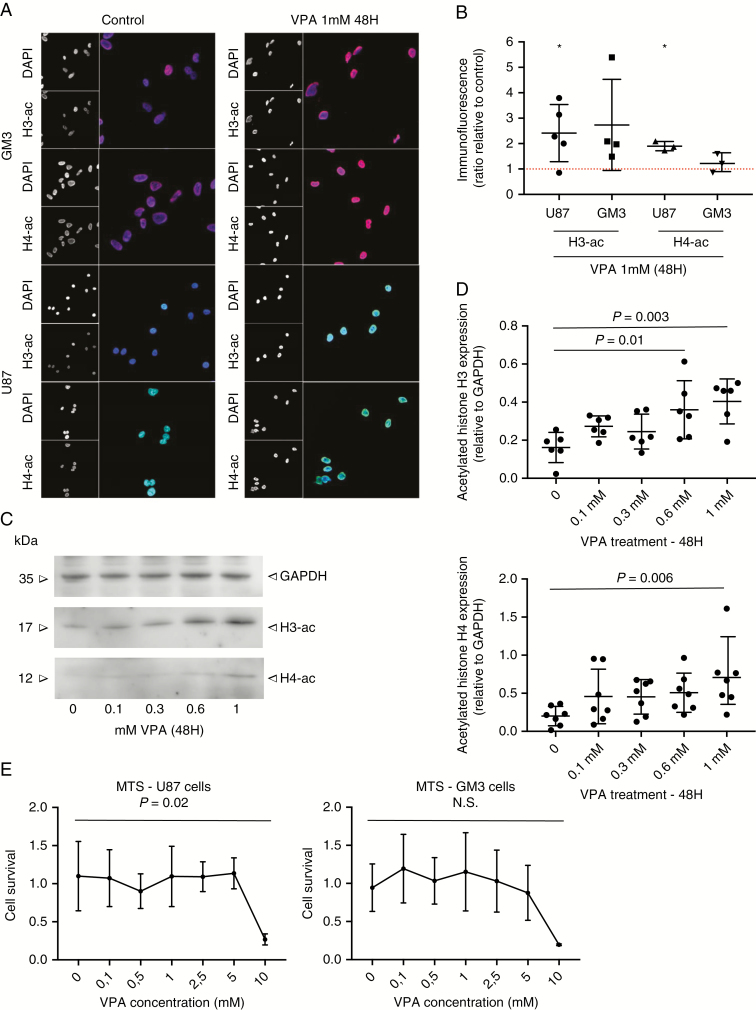
Increased expression of acetylated histone H3 and H4 in GBM cells after VPA treatment. (**A**) Immunofluorescent stainings of acetylated histone H3K9 and H4K8 in GM3 (upper panels) and U87 cells (lower panels). Cells were treated with VPA 1 mM or control during 48 hours. (**B**) Quantification of immunofluorescence experiments shown in (A). Results are shown as ratio compared to control. Bars represent mean ± SD. *n* = 3–5. Results were analyzed by one-sample *t* tests. *P < .05. (**C**) Western blot results for acetylated histone H3K9 (upper panels) and H4K8 (lower panels) and GAPDH (loading control) in U87 cells. Cells were treated with 0, 0.1, 0.3, 0.6, and 1 mM VPA during 48 hours. (**D**) Quantification of western blot results for acetylated histone H3K9 and H4K8, relative to GAPDH expression, in U87 cells. *N* = 6–7. Bars represent mean ± SD. Results were analyzed by two-way ANOVA controlling for interexperimental variation. (**E**) Cell survival assay (MTS test). The proportion of surviving cells after treatment with VPA with increasing dosages (0, 0.1, 0.5, 1, 2.5, 5, 10 mM) for 48 hours is plotted. *N* = 3. Bars represent mean ± SD. Results were analyzed with one-way ANOVA with Dunnet’s post hoc test. GBM, glioblastoma; VPA, valproic acid.

In U87 cells, we confirmed with western blotting and immunocytochemistry that VPA, starting at a concentration of 0.6 mM, can increase histone H3 and H4 acetylation (Fig. 1). In GM3 primary GBM cells we repeatedly observed a similar trend, although these results did not reach statistical significance (Fig. 1 and Supplementary Figure 1). Acute cell toxicity by VPA treatment for 48 hours was only observed with a dosage of 10 mM (Fig. 1E).

### VPA Does Not Increase Histone Acetylation Activity in Tumor Tissue From GBM Patients

Among the 286 patients of our TMA, 29 patients were treated with VPA for epilepsy at the time of their surgery. The control group consisted of 14 GBM patients with epilepsy who did not receive antiepileptic treatment. Staining could not be analyzed for one (histone H3) and two (histone H4) patients from the VPA group, due to insufficient tissue quality on the TMA. The baseline characteristics of these patients are summarized in Supplementary Table 1.

**Fig. 2.**
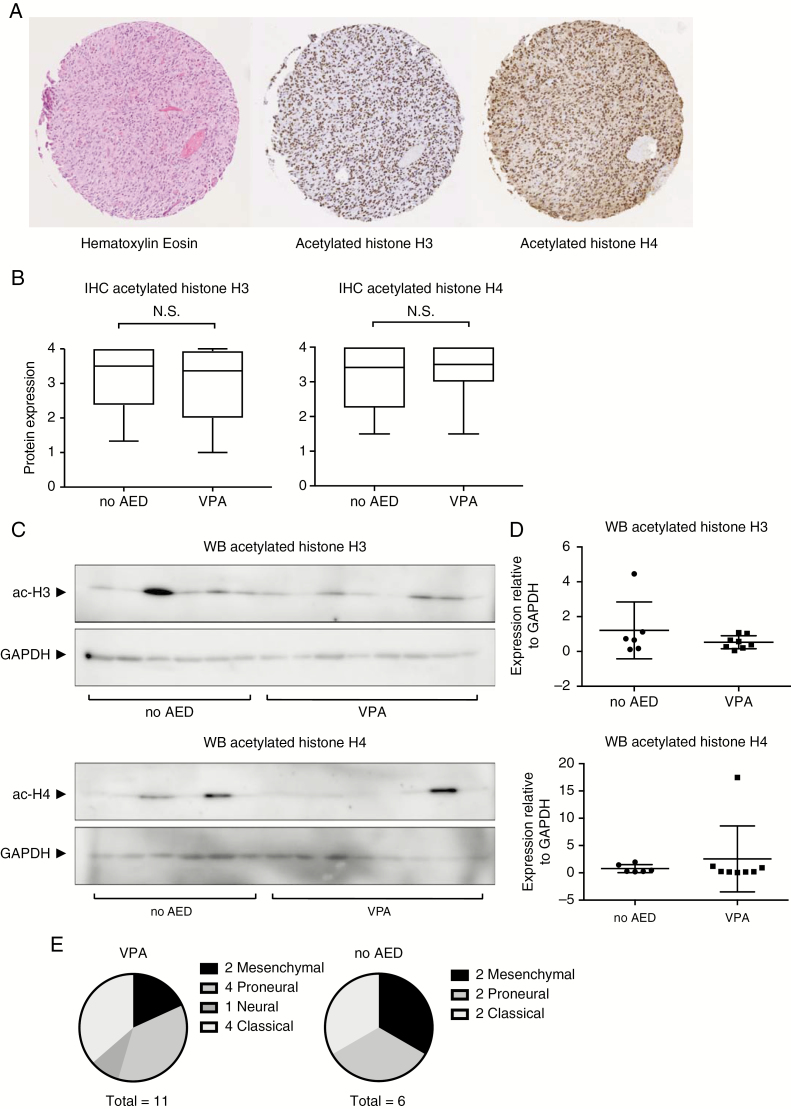
VPA does not increase histone acetylation activity in tumor tissue from GBM patients. (**A**) Examples of immunohistochemical staining of glioblastoma sample on tissue microarray (TMA). The left panel displays hematoxylin and eosin staining, the middle panel shows immunostaining of acetylated histone H3, and the right panel displays immunostaining of acetylated histone H4 in the same patient. (**B**) Quantification of immunohistochemical staining on TMAs containing tumor tissue from glioblastoma patients. TMA’s were stained for acetylated histone H3K9 and H4K8. Boxes represent median and quartiles, whiskers represent data range. Results were analyzed by Mann–Whitney *U* test. (**C**) Quantification of western blot results for acetylated histone H3K9 and H4K8, relative to GAPDH expression, in fresh-frozen patient-derived glioblastoma tissue. Bars represent mean ± SD. VPA: patients that were treated with VPA for epileptic seizures, *n* = 8. No AED: patients had tumor-associated seizures but were not treated with any AED at the time of surgery, *n* = 6. Results were analyzed by Mann–Whitney *U* test. (**D**) Western blot results for acetylated histone H3K9 and H4K8, and GAPDH (loading control) in fresh-frozen GBM tissues. (**E**) Molecular subtype distribution in the patients with VPA and patients with epilepsy but without antiepileptic drugs treatment. Fisher exact test, *P* = 1.0. AED, antiepileptic drug; GBM, glioblastoma; VPA, valproic acid.

There was no difference in the acetylation patterns on TMAs of histone H3 and H4 in the patients treated with VPA as compared to the patients with epilepsy but without any AED (Fig. 2A, Mann–Whitney *U* test, *P* = .41 and 0.46, respectively). In addition, we analyzed acetyl-H3 and acetyl-H4 levels in 14 fresh-frozen GBM samples with western blotting, which might be more sensitive to pick up a difference in histone acetylation levels. Again, there was no difference in acetylated histone H3 and H4 expression between patients that were treated with VPA (*n* = 8) and those that had epileptic seizures, but were not treated with any AED at the time of surgery (*n* = 6, Fig. 2B and C, Mann–Whitney *U* test, *P* = .8 for both experiments).

### VPA Does Not Alter mRNA or Gene Set Expression in GBM Tissue

Affymetrix U133 plus 2.0 RNA expression data were obtained for 12 GBM patients who were treated with VPA at the time of their surgery and for 7 GBM patients who experienced seizures at presentation of the disease, but were not treated with any AED at the time of surgery. Baseline characteristics of these patients are summarized in Supplementary Table 2. Distribution of the molecular subtypes (according to Verhaak et al.^21^) did not differ between the subgroups (Fig. 2D, Fisher exact test, *P* = 1.0). There was no significant mRNA expression difference between in the VPA group compared to the patients without any AED after correction for multiple testing (ANOVA, cutoff *P* < .05, FDR < 0.05). Exploratory GSEA likewise did not show any association between VPA and gene set expression with cutoff *P* < .05 and FDR < 0.1. We did observe an isolated higher expression of the BIOCARTA_AHSP_PATHWAY in the VPA group as compared to patients without any AED (*P* < .0001), but with higher FDR (0.12).

## Discussion

Our in vitro observation that VPA increases histone H3 and H4 acetylation in GBM (primary) cells at concentrations above 0.6 mM after treatment for 48 hours confirms a wealth of previous reports^4,7,9,26,27^ that have triggered the study of use of VPA as an adjunct for the treatment of GBM patients. Both the resulting clinical trials and several retrospective cohort analyses have, however, failed to show any survival benefit of this drug for GBM patients.^12,13^

In order to assess whether VPA has relevant antitumor effects in clinical conditions, we compared the histone acetylation and RNA expression profiles of patient-derived GBM tissue samples obtained from patients treated or not with this drug.

As epileptogenic tumors differ biologically from those that do not elicit epilepsy,^14–17^ we only included GBM patients in our analyses who had epileptic seizures at presentation of the disease. Some other AEDs, for example, topiramate and a major metabolite of levetiracetam, have been reported to influence HDAC expression and function as well.^28,29^ Therefore, in order to avoid any confounding effect, we only included patients in the control group who experienced one or more epileptic seizures but who were not yet treated with any AED prior to their surgery. Although Dutch guidelines recommend treatment with AED for focal tumor-related epilepsy as a rule, the option of withholding AED, for example, after a single seizure, is mentioned as a matter of shared decision making with the patient. Therefore, we could include a (small) control group of patients with epilepsy who did not receive any AED.

In these comparisons, the level of H3 and H4 acetylation did not differ between GBM tissue obtained from patients with seizures that were treated with VPA at the time of their surgery. Likewise, there was no difference of gene expression between these tumors.

A likely explanation for these observations stems from the pharmacokinetics of VPA. Indeed, although serum VPA concentration of 0.7–1.4 mM can be attained with standard clinical dosages of 900–1000 mg VPA twice daily, VPA concentrations in organ tissues, including brain tissue, are eight to nine times lower.^30^ Tissue concentrations are likely to be higher in GBMs as they are characterized by disruption of the blood-brain barrier,^31^ but are unlikely to be equal to those of the serum, and superior to the in vitro threshold of 0.6 mM.

Another potential explanation for the discrepancy between experimental and clinical results is that long term treatment (30 days) has been shown to induce resistance of GBM cells to VPA.^32^ In our study, the median duration of VPA treatment was 33 days, and resistance to GBM cells could not be excluded. This duration of treatment possibly results from the time to referral to our tertiary center. Unfortunately, as a limitation of our retrospective study design, the serum and tissue concentrations of VPA were not available.

In line with the lack of clinical effect of VPA on histone acetylation in GBMs, GSEA showed no associations of VPA on gene set expression in all analyses, except for an isolated higher expression of the BIOCARTA_AHSP_PATHWAY in the VPA group as compared to patients without any AED (*P* < .0001), but with higher FDR (0.12). VPA and other HDACs have been investigated for effects on hemoglobinopathies in the past and a recent preclinical study showed that 0.5 mM VPA treatment could increase Alpha Hemoglobin Stabilizing Protein (AHSP) expression in a human erythroleukemia cell line.^33^

A limitation to our study is that in order to exclude confounding by biological effects due to the presence of epilepsy itself or that of other AEDs, despite a large surgical load at our institution, we could only include a relatively low number of patients. Although larger studies may be necessary to prove the lack of any subtle effect of VPA on the gene expression in tumor tissue in GBM patients, we believe that our findings can seal the discussion on the lack of effect of VPA on the histone acetylation of GBM in the clinic at common dosages.^12,13^

This discrepancy between experimental results and effects in patient samples might explain the lack of effect of VPA on survival of GBM patients. Supraclinical dosages might be necessary to reach the antitumor effects that have been shown in vitro. Alternatively, other methods of HDAC inhibition may prove more relevant.

## Supplementary Material

vdz025_suppl_Supplementary_Figure_S1

vdz025_suppl_Supplementary_Figure_Legends

vdz025_suppl_Supplementary_TableS1

vdz025_suppl_Supplementary_TableS2
