# Supplementary figures and images for: Effects of valproic acid on histone deacetylase inhibition in vitro and in glioblastoma patient samples
Source: Neurooncol Adv. 2019 Nov 12;1(1):vdz025. doi: 10.1093/noajnl/vdz025 (PMC7212905; doi:10.1093/noajnl/vdz025)

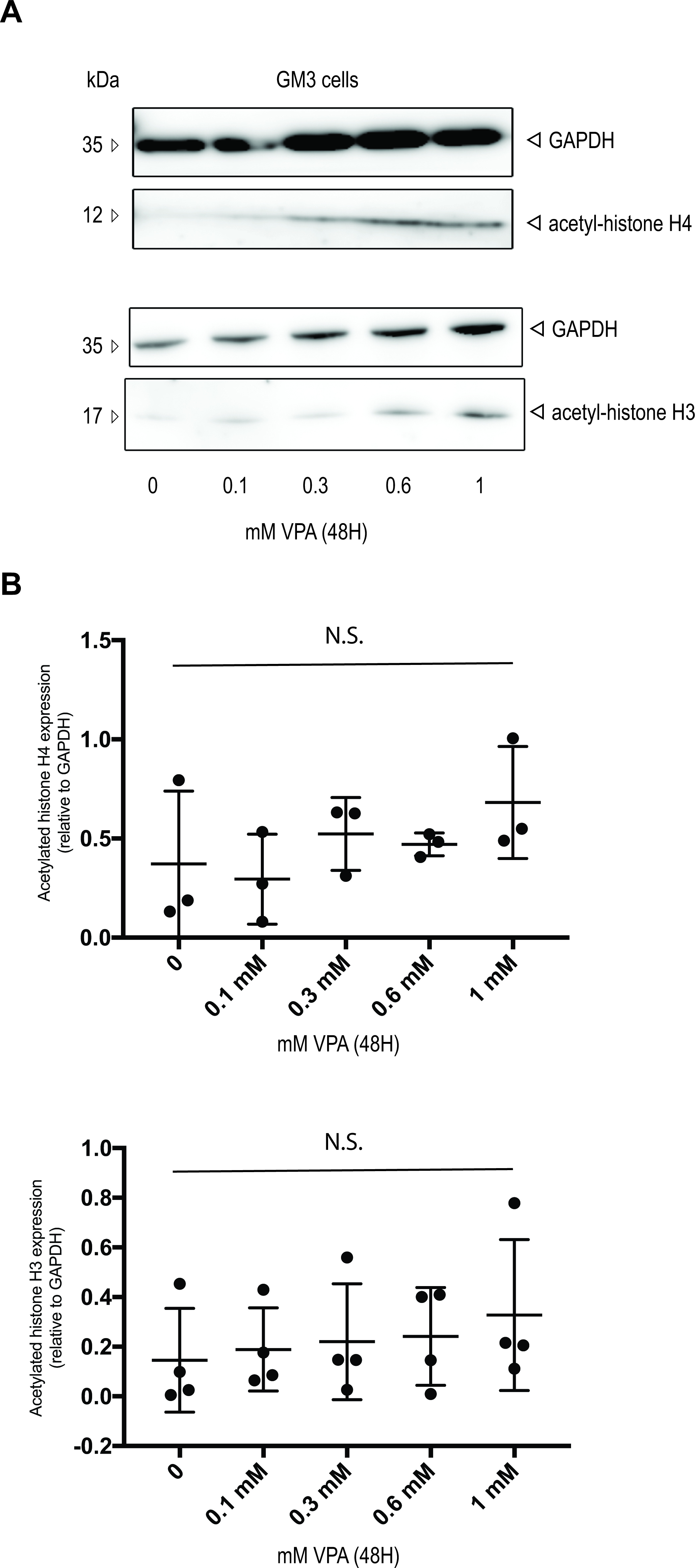

Supplement: vdz025_suppl_Supplementary_Figure_S1 [file vdz025_suppl_supplementary_figure_s1.jpeg]
