# Supplementary material for: Effects of valproic acid on histone deacetylase inhibition in vitro and in glioblastoma patient samples
Source: Neurooncol Adv. 2019 Nov 12;1(1):vdz025. doi: 10.1093/noajnl/vdz025 (PMC7212905; doi:10.1093/noajnl/vdz025)
Supplement: vdz025_suppl_Supplementary_Figure_Legends [file vdz025_suppl_supplementary_figure_legends.docx]

**Supplementary figure legend**

**Figure S1 – Effect of VPA on histone acetylation in GM3 cells.**

1. Western blot results for acetylated histone H3K9 and H4K8 and GAPDH (loading control) in GM3 cells. Cells were treated with 0, 0.1, 0.3, 0.6 and 1 mM VPA during 48 hours.
2. Quantification of western blot results for acetylated histone H3K9 and H4K8, relative to GAPDH expression, in GM3 cells. Bars represent mean ± SD. N=3-4. Results were analyzed by two-way ANOVA controlling for interexperimental variation.
