# Supplementary material for: Effects of valproic acid on histone deacetylase inhibition in vitro and in glioblastoma patient samples
Source: Neurooncol Adv. 2019 Nov 12;1(1):vdz025. doi: 10.1093/noajnl/vdz025 (PMC7212905; doi:10.1093/noajnl/vdz025)
Supplement: vdz025_suppl_Supplementary_TableS1 [file vdz025_suppl_supplementary_tables1.docx]

Table S1 – Baseline table TMA analyses

| Patient characteristics  *n (%)* | Epilepsy without AED  *14 (32.6)* | VPA  *29 (67.4)* |
| --- | --- | --- |
| Age *(mean ± SD)* | 61.1 ± 14.8 | 57.2 ± 12.9 |
| Gender *(% male)* | 64.2 | 58.6 |
| KPS n (%)  < 70  > 70 | 4 (28.6)  9 (64.3)  *Missing: 1 (7.1)* | 8 (27.6)  21 (72.4) |
| Tumor volume *cm^3^*  *(median (range))* | 37.4 (2.5-85.3) | 32.0 (3.1-155.7)  *Missing: 3 (3.2)* |
| Extent of surgery *n (%)*  Biopsy  Debulking | 0  14 (100) | 2 (6.9)  27 (93.1) |
| Post-surgical treatment *n (%)*  None  Monotherapy RT or TMZ  RT + TMZ | 2 (14.3)  2 (14.3)  10 (71.4) | 2 (6.9)  4 (13.8)  23 (79.3) |
| Epilepsy at presentation *n (%)* | 14 (100) | 29 (100) |
| AED treatment *n(%)*  VPA  LEV  CBZ  PHN  Other | -  -  -  -  - | 29 (100)  4 (13.8)  2 (6.9)  2 (6.9)  1 (3.4) |
| Duration VPA treatment *- days*  *(median (range))* | - | 33 (13-196)  *Missing 10 (34.5)* |
| IDH1 mutational status *n (%)*  Wildtype  IDH1 R132H mutation | 14 (100)  0 | 24 (82.8)  3 (10.3)  Missing: 2 (6.9) |

*Abbreviations: VPA: valproic acid; LEV: levetiracetam; CBZ: carbamazepine; PHN: phenytoin; AED: anti-epileptic drugs; KPS: Karnofsky performance score; IQR: interquartile range; RT: radiotherapy; TMZ: temozolomide*
