# Supplementary material for: Effects of valproic acid on histone deacetylase inhibition in vitro and in glioblastoma patient samples
Source: Neurooncol Adv. 2019 Nov 12;1(1):vdz025. doi: 10.1093/noajnl/vdz025 (PMC7212905; doi:10.1093/noajnl/vdz025)
Supplement: vdz025_suppl_Supplementary_TableS2 [file vdz025_suppl_supplementary_tables2.docx]

**Table S2 – Baseline table GSEA samples**

| Patient characteristics  *n (%)* | Epilepsy without AED  *7 (36.8)* | VPA  *12 (63.2)* |
| --- | --- | --- |
| Age *(mean ± SD)* | 58.7 ± 14.6 | 58.6 ± 10.4 |
| Gender *(% male)* | 71.4 | 75 |
| KPS n (%)  < 70  > 70 | 0  7 (100) | 2 (27.6)  10 (72.4) |
| Extent of surgery *n (%)*  Biopsy  Debulking | 0  7 (100) | 0  12 (100) |
| Post-surgical treatment *n (%)*  None  Monotherapy RT or TMZ  RT + TMZ | 0  1 (14.3)  6 (85.7) | 1 (8.3)  1 (8.3)  10 (83.3) |
| Epilepsy at presentation *n (%)* | 7 (100) | 12 (100) |
| Duration VPA treatment *- days*  *(median (range))* | - | 33 (13-146)  *Missing: 2* |
| IDH1 mutational status *n (%)*  Wildtype  IDH1 R132H mutation | 5 (71.4)  0  *Missing: 2 (28.6)* | 5 (41.7)  1 (8.3)  Missing: 6 (50) |

*Abbreviations: VPA: valproic acid; AED: anti-epileptic drugs; KPS: Karnofsky performance score; RT: radiotherapy; TMZ: temozolomide*
